# Can Global Value Chains Embedment Reduce Carbon Emissions Embodied in Exports?—Empirical Test Based on the Manufacturing Industries

**DOI:** 10.3390/ijerph192416458

**Published:** 2022-12-08

**Authors:** Hongwei Zhou, Yawen Kong, Shuguang Liu, Shan Feng

**Affiliations:** 1 School of Economics, Ocean University of China, Qingdao 266100, China; 2 Institute of Ocean Development, Key Research Base of Humanities and Social Sciences, Ministry of Education, Qingdao 266100, China

**Keywords:** manufacturing, global value chains, carbon emissions embodied in exports, energy conservation and emissions reduction

## Abstract

Active participation in the global value chains (GVC) has been recognized as an important factor in curbing the growth of carbon emissions. However, how GVC embedment affects carbon emissions in economies and what are the pathways of its impact need to be further studied. This paper analyzes the mechanism of GVC embedment affecting carbon emissions embodied in exports (CEEE) and selects 17 manufacturing industries in 36 economies around the world for empirical testing. It is found that GVC embedment significantly reduces the CEEE. Specifically, GVC embedment has a suppressive effect on the CEEE of both developed and developing countries, and the former has a greater suppressive effect than the latter; the effect on the CEEE of low-tech industries is significantly negative but not conducive to carbon emissions reduction in high-tech industries; complex and forward embedment have higher emissions reduction effects compared with simple and backward embedment. More importantly, GVC embedment reduces the CEEE through energy conservation effect, structure effect and transfer effect, and all of them show significant inverted U-shaped mediation effect. The findings of this paper have important implications for the sustainable economic development around the world under the GVC division of labor system.

## 1. Introduction

Carbon emissions, as a major factor of climate change, have seriously affected the economics, environment and public health around the world. Recently, carbon emissions embodied in trade (CEET) acts as the key component of carbon emissions, which has increased dramatically. According to the OECD database (OECD database: https://stats.oecd.org/Index.aspx?DataSetCode=TIVA_2021_C1, accessed on 1 December 2022), gross exports around the world increased from 5.77 trillion USD to 21.73 trillion USD with an increase of 276.34%, and carbon emissions embodied in exports (CEEE) rose from 5103.2 Mt to 9697.7 Mt with an increase of 90.03% during the period of 1995–2018. Thus, the study of how to reduce CEET is crucial to realize the targets of sustainable development goals (SDGs) and the Paris Agreement.

In the past two decades, the international division of labor system based on global value chains (GVC) has become the typical feature of economic globalization, which has a significant impact on CEET (Liu, 2020) [1]. As a global network, it connects the production processes in different countries to obtain the value-added from goods and services (Liu et al., 2018) [2]. An increasing number of countries, especially developing countries, have participated in GVC due to the benefits of expanding new markets, obtaining knowledge spillover and enhancing technology innovation level in environment (Song and Wang, 2017) [3]. Meanwhile, there are substantial variations in terms of benefits and costs of GVC embedment between developed and developing countries when they have different positions. Specifically, developed countries are mainly engaged in high value-added and low-pollution activities, tending to transfer high-pollution activities to other countries. In contrast, developing countries usually undertake high-pollution industries from developed countries so as to embed in GVC (Huang, 2022) [4]. Then, does the improvement of the GVC embedment position contribute to the reduction of CEET? What is the mechanism of its impact? Is there any difference in the role of the degree of GVC embedment on CEET reduction for economies at different development levels? Clarifying the above questions is crucial for countries to formulate targeted carbon emissions reduction policies and promote the achievement of carbon neutrality targets.

This paper aims to clarify how GVC embedment affects the CEEE and identify its impact pathways, which fills the existing research gaps on carbon emissions. Firstly, we construct a uniform framework to analyze GVC embedment and CEEE and explore three paths of the former’s influence on the latter, which is described by the energy conservation effect, structure effect and transfer effect. Secondly, based on the mediating effects model, energy consumption intensity, energy consumption structure and carbon emissions transfer are selected as mediators to examine these paths. Thirdly, based on the global perspective, we select the data (The data for the WIOD database environmental accounts are from 2000–2016, so the sample period is also set for this interval in this paper; The WIOD database contains 43 economies, and due to data availability constraints, the exclusion of Chile, Cyprus, Luxembourg, Latvia, Malta, Russia, and Taiwan, China yields a total of 36 economies, which together account for more than 80% of the global GDP; The OECD industry codes are: D10T12, D13T15, D16, D17T18, D19, D20, D21, D22, D23, D24, D25, D26, D27, D28, D29, D30, D31T33; WIOD industry codes are: C10-C12, C13-C15, C16, C17, C18, C19, C20, C21, C22, C23, C24, C25, C26, C27, C28, C29, C30, C31_C32) of 17 manufacturing sectors in 36 major economies around the world from 2000–2016 as research samples to conduct the empirical test. This research provides specific suggestions for energy conservation and emissions reduction of economies in different GVC positions. The remainder of this study is as follows. Section 2 reviews the relevant literature. Section 3 describes the theoretical mechanism. Section 4 analyzes the core indicators metrics and status. Section 5 introduces the empirical model, estimation method, variables description and data source. Section 6 shows the empirical results including baseline regression, heterogeneity analysis, robustness test and mechanism test results. Finally, conclusions, discussions and limitations of the research are drawn in Section 7 and Section 8.

## 2. Literature Review

### 2.1. Concept and Measurement of GVC

The GVC, originated from the vertical specialization theory proposed by Balassa in 1967, emphasized that each country specializes in a specific stage of production and the parts of product production were connected through trade chains (Balassa, 1967; Grubel, 1975; Findlay, 1978) [5,6,7]. Since the 1980s, scholars have improved GVC in different perspectives. For example, Porter (1985) [8], Krugman (1995) [9] and Sturgeon (2001) [10] elaborated the definition and characteristics of GVC based on value system, value-added and spatial reorganization, respectively. Gereffi (1994) [11] incorporated industrial organization and global spatial allocation into the research framework and figured out the connection between GVC and globalized economy.

On the basis of GVC, the embeddedness of GVC was measured from different perspectives. For example, based on the vertical specialization division of labor, Hummels (2001) [12] measured a country’s GVC participation by the foreign value-added rate of export trade (VSS), which was improved by the value-added return index (Daudin, 2011) [13] and the weight index (Johnson, 2012) [14]. However, this method underestimated the value of GVC embedment without considering the domestic value added in exports of intermediate goods. Fally (2012) [15] used the production segmentation length to represent the production length of GVC. Based on input-output tables, Antras (2012) [16] and Antras and Chor (2013) [17] proposed upstream and downstream value chains index to reflect a country’s GVC participation, respectively. Koopman (2014) [18] established the KWW framework to decompose export trade flows into four components: domestic value-added absorbed abroad; domestic value-added returned to the country; foreign value-added; and double counting. Wang et al. (2013) [19] established the WWZ framework, followed by the WWYZ framework (Wang et al., 2017; 2017) [20,21], which proposed an indicator system that reflected the position and participation in the GVC division of labor more accurately.

### 2.2. The Determinants of Carbon Emissions Embodied in Trade

As one of the most dominant activities in the international division of labor, the scales and sources of carbon emissions generated in international trade have gained continuous attention from scholars. The existing studies have mainly focused on the following three aspects. The first is the measurement of CEEE indicators. Zhao (2011) [22] measured the CEEE based on the single-regional input-output method. This method overestimated the coefficient of carbon emissions without considering the influence of imported intermediate products. With the continuous updating of global input-output databases such as WIOD, Eora and EXIOBASE, the measurement method evolved into a multi-regional input-output model (Qiao, 2018; Tang and Zhu, 2022) [23,24], and the estimation perspective transferred from gross trade to more precise value-added trade (Cheng, 2020) [25]. The second is the influence factors of CEEE. Li (2010) [26] believed that direct emissions coefficient, production technology, export scale and export structure effect factors affected China’s CEET, and the direct emissions coefficient factor is the main growth-promoting factor (Yin, 2019) [27]. Utilizing the Logarithmic Mean Divisia Index (LMDI) method to structurally decompose China’s carbon emissions embodied in trade, Wang (2011) [28] argued that the main factors contributing to China’s net transfer of CEET are intensity effect, scale effect and structure effect. With the deepening of foreign direct investment (FDI) and vertical specialization division of labor, the structural change of FDI industry (Li, 2012) [29] and the vertical division of labor system in East Asia (Qian, 2016) [30] gradually dominated the growth of China’s CEET. The improvement of green production technology efficiency effectively suppressed the growth of China’s carbon emissions embodied in value-added exports (Fu, 2018; Li et al.,2022) [31,32]. The last is the attribution of responsibility for emissions. Based on data from 28 countries of OECD. Wang (2011) [33] found that the developing countries bear a large amount of carbon emissions for consumers in developed countries during international trade activities (Yan, 2012) [34]. In addition, some scholars explored the transfer trends of CEET from the perspective of bilateral trade (Liu, 2017; Meng, 2019; Wang, 2019; Li, 2020) [35,36,37,38], which revealed the relationship between international trade and the environment from the dual perspective of trade gains and environmental costs and proposed policy measures to conduct bilateral trade for China in the future.

### 2.3. The Environmental Effects of GVC in Trade

In recent years, scholars engaged in an intense debate on the relationship between the GVC and CEEE. In terms of GVC embedment, Lv (2017) [39] argued that forward embedment of GVC may significantly reduce the carbon emissions embodied in China’s industrial sector exports, while the result for backward embedment is opposite. Zhao (2021) [40] analyzed 56 industry sectors in China and drew a different conclusion, suggesting that both forward and backward embedment are not conducive to reducing CEEE. Chen (2022) [41] showed that forward embedment will increase the forward production chain length and improve the GVC position index compared to backward embedment, which may reduce the CEEE in labor-intensive and technology-intensive industries. Besides, Lv (2019) [42] argued that there is a non-linear relationship between GVC participation and China’s CEEE and proposed that GVC participation has a double threshold characteristic with technology level as a smoothing transformation variable for carbon emissions embodied in exports, imports and trade balance. In terms of GVC position, Pan (2020) [43] and Wang et al. (2022) [44] found that lower GVC position causes higher CEEE of China, which expands as GVC position gradually deepens. Liu (2020) [45] argued that there is a negative relationship between GVC position and CEEE, in which the GVC effect exceeds the size, structure and technology effects.

## 3. Theoretical Analysis

As international vertical specialization continues to deepen, it has become the consensus of all countries to facilitate economic development by actively integrating into GVC, while the carbon emissions generated in this process have expanded rapidly. The proper reason is that GVC embedment of one country increases its demand for energy in the production process, and thus the carbon emissions expand. Improving the GVC embedment reduces the carbon pollution caused by international division of labor. On one hand, the increase in the GVC embedment is beneficial for enterprises to improve the efficiency of energy use. Host countries obtain technology spillover to improve technology (Su, 2017) [46] and industry productivity (Lv, 2017) [47], which reduces energy consumption per unit of output. On the other hand, deep GVC embedment may prompt both partners to develop standardized industrial policies (Gereffi, 2013) [48] and trade policies (Sheng, 2015) [49], which in turn promote the industry to optimize production and trade structures and reduce non-clean energy use and carbon emissions. More importantly, enterprises embedding in GVC can make full use of the advanced knowledge elements in the GVC to move up the higher position (Bai, 2022) [50]. When a country climbs up to a higher position of the GVC, it will undertake non-productive links with high value-added and low-energy consumption in the international division of labor such as R&D, design and brand operation. While the low value-added and high energy-intensive links of the country such as transport, production are transferred to countries at the lower end of the GVC, thus achieving high trade gains and low environmental costs. Based on the above analysis, this paper proposes the Hypothesis 1.

**Hypothesis** **1 (H1).** *Increasing the GVC embedment reduces the CEEE*.

**Figure 1:**
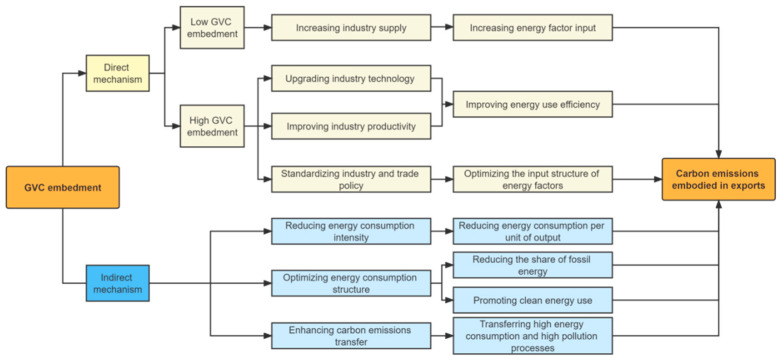
Theoretical mechanism of the impact of GVC embedment on the carbon emissions embodied in exports.

Then, how does the GVC embedment affect the carbon emissions generated in a country’s export activities? This paper will explore the impact paths of GVC embedment on the intensity of carbon emissions in exports (ICEEE) from three aspects: energy consumption intensity; energy consumption structure; and carbon emissions transfer (see Figure 1).

Firstly, energy consumption is the main source of pollutant emissions in the production process (Akhmat, 2014) [51], and the GVC embedment will cut down the energy consumption per unit of output to some extent. For one thing, the GVC division of labor system promotes the effective flow and integration of heterogeneous production factors, improves resource allocation and factor-output efficiency and reduces the proportion of energy factor inputs. For another, through technology spillover or reverse technology spillover obtained by bilateral direct investment, international trade in products and services, and patent transfer, countries embedding in GVC improve the technological level of industry production and export products (Wang, 2019) [52] and total factor productivity (Su, 2020) [53]. Both the increase in technology and productivity will improve the industry energy use efficiency and reduce the energy consumption per unit of output to reduce carbon emissions intensity. In addition, GVC, as a carrier for the human capital, intellectual capital and green innovation technology elements (Liu, 2021) [54], is conductive to promoting technological change, factor input optimization and production model transformation in the industry to lower the intensity of energy consumption and pollutant emissions. Therefore, GVC embedment inhibits CEEE by improving energy utilization efficiency and reducing energy consumption intensity of the industry. Based on the above analysis, this paper proposes the Hypothesis 2.

**Hypothesis** **2 (H2).** *GVC embedment has an energy conservation effect that will inhibit the CEEE by reducing the energy consumption intensity*.

Secondly, GVC embedment increases the proportion of clean energy elements in the production process to optimize the energy consumption structure and then reduces the CEEE. Industries at the top of the GVC are mainly engaged in high value-added R&D and design activities, which effectively drive clean technology innovation and progress to improve clean energy utilization efficiency and reduce carbon emissions intensity. Besides, on the one hand, industries with a higher position in GVC can jointly set high standards of product production and trade environmental guidelines in order to force enterprises to adjust the ratio of energy inputs and lower the non-clean energy use including coal. On the other hand, they have been increasing R&D investment to improve technology to optimize the energy consumption structure and decrease carbon emissions intensity. To sum up, GVC embedment optimizes the energy consumption structure by innovating clean technology, improving environmental protection standards and technology level, thus reducing the CEEE. Based on the above analysis, this paper proposes the Hypothesis 3.

**Hypothesis** **3 (H3).** *GVC embedment has a structure effect that will reduce the CEEE by optimizing the energy consumption structure*.

Thirdly, based on the factor endowment theory, GVC division of labor system will cause a global transfer of carbon emissions (Copeland and Taylor, 1997) [55]. Countries with comparative advantage in energy factor division of labor focus more on high value-added and low-pollution services such as R&D and distribution in the process of international division of labor and transform non-clean production processes with high energy consumption and high pollution to other countries, thus reducing domestic carbon emissions. Therefore, GVC embedment enhances carbon emissions transfer by transferring high pollution production links, thus reducing the CEEE. Based on the above analysis, this paper proposes the Hypothesis 4.

**Hypothesis** **4 (H4).** *GVC embedment has a transfer effect that will reduce the CEEE by enhancing carbon emissions transfer*.

## 4. Core Indicators Metrics and Status Analysis

### 4.1. Core Indicators Metrics

#### 4.1.1. Degree of GVC Embedment

The degree of GVC embedment is the core explanatory variable in this paper, which is characterized by GVC participation index ($GVC\_Pat_{ijt}$) based on the WWYZ (2017) [21] method.

(1) $$\begin{array}{ll} GVC\_Pat\_f & =GVC\_Pat\_f\_s+GVC\_Pat\_c\\ & =VAsgvc/SVA+VAcgvc/SVA \end{array}$$

(2) $$\begin{array}{ll} GVC\_Pat\_b & =GVC\_Pat\_b\_s+GVC\_Pat\_b\_c\\ & =FVAsgvc/FG+(DVAcgvc+FVAcgvc/FG) \end{array}$$

Here, GVC_Pat_f is the forward participation index of global value chain, and GVC_Pat_b is the backward participation index. GVC_Pat_f_s and GVC_Pat_c are the forward participation index of simple GVC embedment and complex GVC embedment, respectively. GVC_Pat_b_s and GVC_Pat_b_c are the backward participation index of simple GVC embedment and complex GVC embedment, respectively. SVA is the value-added of the industry. VAsgvc is the value-added embodied in exports of intermediate products directly absorbed by importers (simple GVC activities). VAcgvc is the value-added embodied in exports of intermediate products that are further used by the importer to produce exports (complex GVC activities). FG is the gross output of final products. DVAcgvc is domestic value-added returned and consumed domestically (complex GVC activities). FVAsgvc is partner value-added directly created in production of domestically consumed products (simple GVC activities). FVAcgvc is foreign value-added created in production of final products, except FVAsgvc (complex GVC activities).

**Figure 2:**
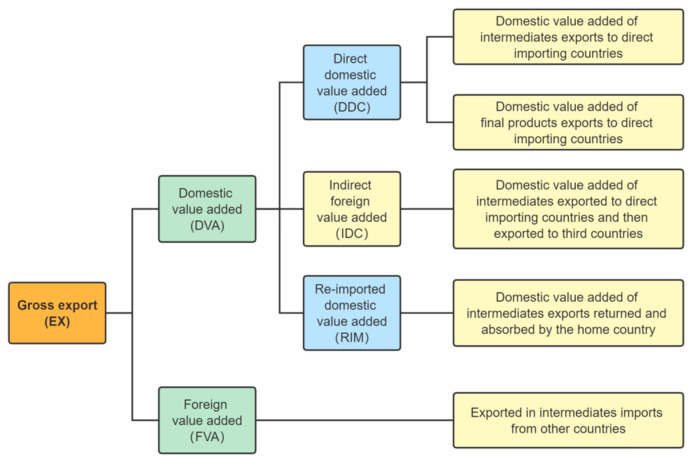
Decomposition of value-added in gross exports based on the OECD database.

On this basis, drawing on Koopman’s (2014) [18] decomposition framework for gross exports (see Figure 2), the industry-level GVC participation index GVC_Pat is as follows:

(3) GVC_Pat=GVC_Pat_f+GVC_Pat_b

#### 4.1.2. Intensity of Carbon Emissions Embodied in Exports (ICEEE)

Value-added of exports and CEEE are reflections of trade gains and environmental costs paid in the international trade process. Therefore, this paper uses the share of domestic carbon emissions to domestic value-added embodied in exports to characterize the ICEEE. Most of the literature has used carbon emissions per unit of gross exports to characterize the ICEEE, which undoubtedly underestimates the actual value. For example, if an Apple cell phone is assembled in China and exported to the world for consumption, the gross export value is calculated as 178.65 USD, while the real trade gains (value-added) to China is calculated as 6.5 USD. Then, using 178.65 USD of exports as the denominator would significantly underestimate the environmental costs of Chinese exporters participating in the GVC. To analyze the environmental costs of exports based on the GVC perspective, we need to compare the “value-added of exports” with the “CEEE”. Value-added of exports can be divided into domestic value-added and foreign value-added, which are subdivided into eight indicators; CEEE can be divided into domestic carbon emissions and foreign carbon emissions, which are subdivided into four components corresponding to value-added of exports (see Table 1).

### 4.2. Status Analysis of the Degree of GVC Embedment

**Figure 3:**
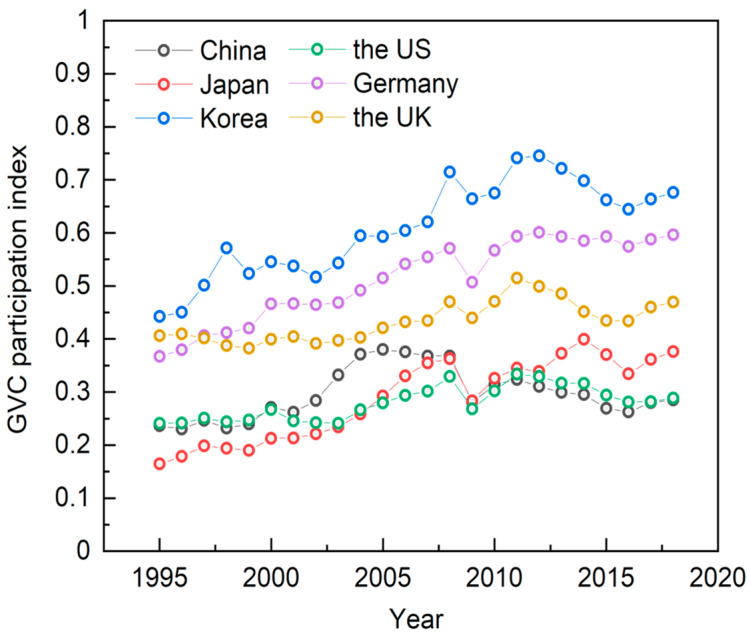
Trends in the GVC participation index of manufacturing in major global economies, 1995–2018. Data source: Authors’ calculations based on UIBE database.

Currently, the international vertical division of labor system tends to mature, and embedding in the GVC has become an important form of participation in the international division of labor for each country. Figure 3 shows the trends of the GVC participation index of manufacturing in major global economies from 1995 to 2018. Longitudinally, the GVC participation index of each economy has increased to different degrees during the sample period and decreased after the financial crisis, with Korea, Germany and the UK always maintaining relatively high levels. Horizontally, GVC participation index of China is still significantly lower than that of major developed economies.

### 4.3. Status Analysis of Carbon Emissions Embodied in Exports

**Figure 4:**
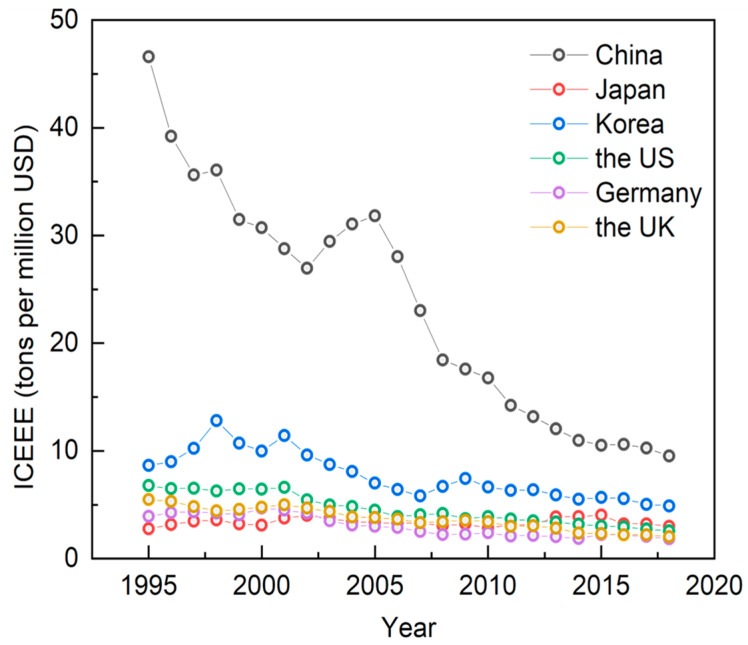
Trends in the ICEEE of manufacturing in major global economies, 1995–2018 (in tons per million USD). Data source: Authors’ calculations based on OECD database.

**Figure 5:**
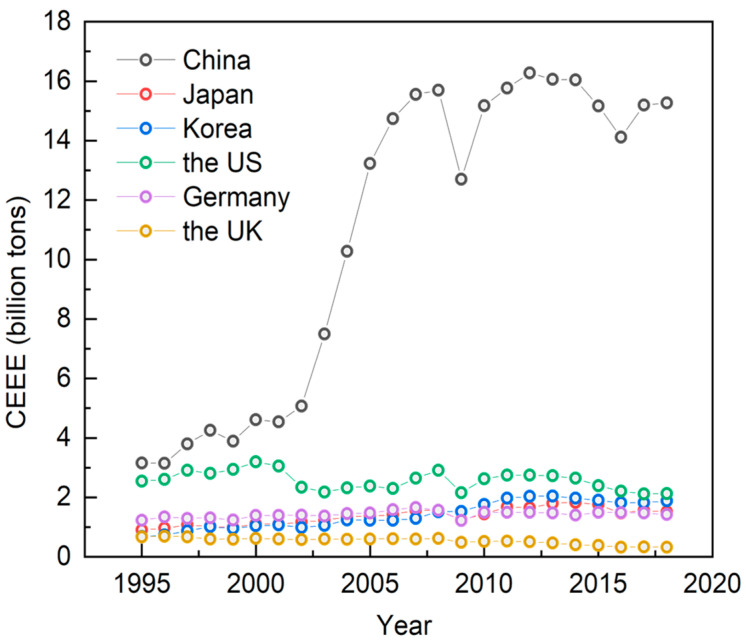
Trends in total CEEE of manufacturing in major global economies, 1995–2018 (in billion tons). Data source: Authors’ calculations based on OECD database.

The total and intensity of carbon emissions embodied in exports reflect the environmental costs paid by a country in the process of participating in the international division of labor. Figure 4 shows the trends of the ICEEE of manufacturing in major global economies from 1995 to 2018. Vertically, all countries have a decreasing trend of the ICEEE to some extent, with Japan, Germany, the U.S. and the U.K. changing more steadily; the ICEEE in China and the U.S. have decreased after the financial crisis. Horizontally, the ICEEE of manufacturing in China is still significantly higher than that of major developed economies. Figure 5 shows the trends of total carbon emissions embodied in exports of manufacturing in major global economies from 1995 to 2018. Vertically, Germany, South Korea, and Japan have changed little; the U.S. shows a significant decrease after the financial crisis but has remained largely stable since then, and the U.K. has a continuous downward trend. Total carbon emissions embodied in exports of manufacturing in China have increased sharply since its access to the WTO in 2001, declined briefly after the financial crisis, but rebounded soon after. Horizontally, total carbon emissions embodied in exports of manufacturing in China are still significantly higher than those of major developed economies. In conclusion, China has always been at a high level in terms of both the total and intensity of carbon emissions embodied in exports of manufacturing, which is under greater pressure on energy conservation and emissions reduction.

## 5. Material and Methods

### 5.1. Model Setting and Estimation Method

#### 5.1.1. Baseline Regression Model 

To empirically test the extent of the impact of GVC embedment on the ICEEE, the following benchmark model is constructed.

(4) $$TEC_{ijt}=\alpha_{0}+\alpha_{1}GVC\_Pat_{ijt}+\alpha_{2}TS_{ijt}+\alpha_{3}ES_{ijt}+\alpha_{4}TFP_{ijt}+\alpha_{5}X_{ijt}+\lambda_{i}+\mu_{j}+\pi_{t}+\varepsilon_{ijt}$$

where i is the 36 countries or regions after excluding part of the sample; j is the 17 sectors of the manufacturing industry, and t denotes the year. $TEC_{ijt}$ is the intensity of carbon emissions embodied in industry j exports of country i in year t. $GVC\_Pat_{ijt}$ is the industry-level GVC participation index, which is used to characterize the degree of GVC embedment and is the core explanatory variable of this paper. $TS_{ijt}$, $ES_{ijt}$ and $TFP_{ijt}$ represent trade size, economic structure and total factor productivity, which characterize the scale, structure and technology effect, respectively. $X_{ijt}$ is the remaining sets of control variables. $\lambda_{i}$, $\mu_{j}$ and $\pi_{t}$ denote country, industry and time fixed effect, respectively. $\varepsilon_{ijt}$ is random error term.

Considering that pollution variables such as carbon emissions have a certain degree of historical dependence, their first-order lagged terms are taken and placed in the model, and a dynamic panel regression model is constructed as follows.

(5) $$TEC_{ijt}=\alpha_{0}+\alpha_{1}TEC_{ijt-1}+\alpha_{2}GVC\_Pat_{ijt}+\alpha_{3}TS_{ijt}+\alpha_{4}ES_{ijt}+\alpha_{5}TFP_{ijt}+\alpha_{6}X_{ijt}+\lambda_{i}+\mu_{j}+\pi_{t}+\varepsilon_{ijt}$$

where $TEC_{ijt-1}$ is the lagged term of the intensity of carbon emissions embodied in industry exports and the rest of the variables are the same as above.

Meanwhile, this paper uses the three-dimensional data of “country-industry-year,” and the model can be estimated by using two-way fixed effects and introducing dummy variables, but its convergence is slow, and it generates relatively large errors. Therefore, in this paper, the multi-way fixed effects estimation method (REGH) is chosen to estimate the above model (Correia, 2016) [57], which can effectively control for country, industry and year fixed effects.

#### 5.1.2. Mechanism Test Model 

To test Hypothesis 2–4, referring to Wen (2004) [58], this paper sets up the mediating effect model as follows.

(6) $$TEC_{ijt}=\alpha_{0}+\alpha_{1}TEC_{ijt-1}+\alpha_{2}GVC\_Pat_{ijt}+\alpha_{3}TS_{ijt}+\alpha_{4}ES_{ijt}+\alpha_{5}TFP_{ijt}+\alpha_{6}X_{ijt}+\lambda_{i}+\mu_{j}+\pi_{t}+\varepsilon_{ijt1}$$

(7) $$Med_{ijt}=\beta_{0}+\beta_{1}Med_{ijt-1}+\beta_{2}GVC\_Pat_{ijt}+\beta_{3}X_{ijt}+\lambda_{i}+\mu_{j}+\pi_{t}+\varepsilon_{ijt2}$$

(8) $$TEC_{ijt}=\gamma_{0}+\gamma_{1}TEC_{ijt-1}+\gamma_{2}GVC\_Pat_{ijt}+\gamma_{3}Med_{ijt}+\gamma_{4}X_{ijt}+\lambda_{i}+\mu_{j}+\pi_{t}+\varepsilon_{ijt3}$$

where $Med_{ijt}$ is mediating factors which characterize the energy conservation effect, structure effect and transfer effect, respectively.

### 5.2. Variables Description

The core variables of this paper have been measured and analyzed above, and to reduce omitted variable bias, this section focuses on the setting of mediators and the description of the set of control variables at both industry and country levels.

#### 5.2.1. Mediators

(1) The energy conservation effect is characterized by reducing energy consumption intensity (EY). Most of the existing literature use energy input per unit of GDP to measure energy consumption intensity. Therefore, this paper uses the share of total energy consumption related to carbon emissions to total industry output to measure energy consumption intensity and deflates total industry output by price index to the sample base period (2000).
(2) The structure effect is characterized by optimizing energy consumption structure (ES). Fossil energy represented by coal and crude oil may produce higher pollutant emissions in the production process, and the higher their share in energy input, the more pollutant emissions they produce. Therefore, this paper uses the share of the consumption of coal and crude oil related to carbon emissions in the total energy consumption to measure energy consumption structure.
(3) The transfer effect is characterized by enhancing domestic carbon emissions transfer (FR). Countries embedding in higher position of GVC may transfer carbon emissions to other countries in lower position, which will reduce their own carbon emissions. Therefore, based on Peng et al. (2015) [59], this paper uses the share of foreign carbon emissions to total carbon emissions embodied in exports to measure carbon emissions transfer.

#### 5.2.2. Control Variables

Industry level. (1) Trade size (TS). Based on Wang et al. (2013) [18], in this paper, domestic value added (DVA) of exports is selected to characterize the scale effect in order to measure the true value of domestic exports created and fully absorbed by foreign countries. (2) Trade structure (ES). The international vertical division of labor system is maturing, and countries that obtain higher value-added in trade activities will continue to stabilize and increase exports of products with comparative advantage. This paper uses the share of domestic value added in total exports as a proxy variable for the structure effect. (3) Total factor productivity (TFP). The improvement of technology level may effectively reduce the proportion of high pollution energy consumption, improve energy efficiency and reduce carbon emissions in the production process (Lv, 2019) [47]. (4) Number of employees (EMP). Industries with a larger number of employees are generally labor-intensive and bear more downstream links such as processing and assembly in the international division of labor, which may generate higher carbon emissions. (5) Output per capita (OPC). The ratio of total output to the number of employees at industry level is chosen to measure it. (6) Trade openness (OPEN). The ratio of industry exports to total output is chosen to measure it. (7) Global value chains position (GVC_Pos). One country in high position of the GVC implies that it mainly produces and exports high value-added and low-polluting products, which will reduce the CEEE. Based on Koopman’s (2014) [15] decomposition framework for gross exports, this paper constructs the GVC position index as follows.

(9) GVC_Pos=ln(1+GVC_Pat_f)−ln(1+GVC_Pat_b)

Country level. (1) Economic size (GDP). The higher the level of economic development, the more links it assumes in the international division of labor, and the more the carbon emissions generated in the production and trade process. This paper uses GDP as the proxy variable of economic size. (2) Scale of foreign direct investment (FDIR). The larger scale of FDI, the more likely the host country becomes a “pollution heaven” and the more the carbon emissions. The share of FDI in GDP is chosen to measure it.

### 5.3. Data Source

The data sources used in this paper are as follows: (1) World input-output tables: the world input-output tables used in this paper are obtained from OECD ICIO database, the latest version of which is the 2021 edition, covering input-output data of 45 industries in 67 economies around the world from 1995 to 2018. (2) Intensity of carbon emissions embodied in exports at industry level: the data of domestic value added are obtained from the OECD TiVA account of 2021 edition, and the data of carbon emissions embodied in exports are obtained from the OECD STAN account of 2021 edition, which are calculated and collated to obtain the ICEEE at industry level. (3) GVC embedment indicators: GVC participation and position index of manufacturing sectors of countries from 2000 to 2016 used in this paper are obtained from the UIBE database, and the original indicators required for the calculation are obtained from the OECD ICIO database. (4) The data of FDI and GDP are obtained from the UNCTAD database and WITS database, respectively, and they are deflated to 2000 according to the price index. The raw data of remaining control variables are obtained from the OECD TiVA database and WIOD environmental account of 2016 edition. Besides, this paper collates and groups the industry codes of the manufacturing sectors in two major databases and finally obtains 17 sub-sectors. Finally, when estimating the industry total factor productivity, the input factors are set as the number of employees in the industry, fixed capital stock and energy consumption related to carbon emissions. The desirable output is the value-added of industry exports, and the undesirable output is carbon emissions embodied in exports.

## 6. Empirical Results 

### 6.1. Baseline Regression Results

To compare the applicability and difference of various measurement methods, we use OLS regression, general static fixed effects, multidimensional static fixed effects and multidimensional dynamic fixed effects to estimate the above model, respectively. Models 1 to 4 in Table 2 show the estimation results which indicate that the R-squared estimated by multidimensional dynamic fixed effects is better than other methods, and the lagged term coefficients of the independent variables are significant at the 1% level. More importantly, the results show that GVC embedment will significantly reduce the ICEEE, and when the degree of GVC embedment increases by 1%, the intensity decreases by about 0.075%.

Meanwhile, in order to verify the possible non-linear relationship between GVC embedment and the ICEEE, referring to Xu and Mao (2016) [60], the sample is divided into high GVC embedment group (High × GVC) and low GVC embedment group (Low × GVC) according to the 50% quantile to examine the differences between groups of emissions reduction effect. Models 5 to 6 in Table 2 show that the negative effect of GVC embedment on the ICEEE is not significant when the degree of GVC embedment is low, while the negative effect of GVC embedment on the ICEEE becomes significant as the degree of GVC embedment increases. Therefore, the Hypothesis 1 is testified.

### 6.2. Heterogeneity Analysis Results

#### 6.2.1. Country Heterogeneity Results

The environmental effects of GVC embedment may vary from countries at different levels of development. According to the World Bank’s classification of income level per capita, the sample is divided into the developed and developing countries. Model 1 in Table 3 shows the country heterogeneity results, which indicate that GVC embedment has a significant inhibitory effect on the ICEEE for both developed and developing countries, and the inhibitory effect of the former is greater than that of the latter. The results indicate that, on one hand, the “low-end locking” that developing countries suffer at the economic level may not necessarily exist at the environmental level; on the other hand, developing countries can absorb advanced green technology by embedding in GVC to lower their CEEE. The increase in GVC embedment in developed countries will enable them to integrate advanced knowledge elements systematically, optimize their production and trade structures and relocate high-polluting industries so that their emissions reduction effect is better than that of developing countries.

#### 6.2.2. Industry Heterogeneity Results

The environmental effects of GVC embedment may vary from manufacturing industries. According to the classification method based on technology intensity released by OECD in 2011, manufacturing industries are classified into four categories: low-tech; medium-low-tech; medium-high-tech; and high-tech. This paper considers low-tech and medium-low-tech as low-tech industries and the other two categories as high-tech industries. Model 2 in Table 3 shows the industry heterogeneity results, which indicate that GVC embedment does not inhibit the ICEEE in high-tech industries. The possible explanation is that firms in high-tech industries prefer to develop low-carbon and clean technologies to improve their energy utilization efficiency (Huang, 2017) [61], resulting in less emissions reduction space. In contrast, GVC embedment has a significant inhibitory effect on the ICEEE in low-tech industries with more space for emissions reduction.

#### 6.2.3. Embedment Methods Heterogeneity Results

The environmental effects of GVC embedment may vary from embedment methods. According to the embedment position, this paper divides the GVC embedment into forward and backward embedment. Moreover, based on the number of cross-border production activities, this paper classifies the GVC embedment as simple embedment (export products are directly absorbed by importing countries without crossing the border again) and complex embedment (export products are directly absorbed by importing countries with crossing the border to third countries again). 

Model 3 in Table 3 shows the embedment methods heterogeneity results, which indicate that forward embedment of GVC will significantly reduce the ICEEE, while the inhibitory effect of backward embedment is not significant; simple embedment of GVC does not reduce and even promote the ICEEE, while complex embedment of GVC has a significant inhibitory effect on the ICEEE.

### 6.3. Robustness Test Results

To examine the robustness of above results, this paper adopts the following methods. First, the independent variables are remeasured. The intensity of carbon emissions embodied in exports (TECT) is remeasured by replacing value added with gross exports as the denominator. Second, this paper uses regression between groups. The sample is further divided into low (D1), medium (D2) and high (D3) GVC embedment based on the trichotomies. Third, endogeneity among variables is considered. The possible two-way causality between GVC embedment and the ICEEE and the possible omission of variables in the model setting process may affect the estimation results, so this paper adopts the IV-2SLS method and IV-DREGH method (a combination of instrumental variables method and multidimensional fixed effects) to estimate the model. The conditions that the instrumental variables should satisfy are: highly correlated with GVC embedment and uncorrelated with the random error term. Therefore, the first-order lagged term of GVC embedment is selected as the instrumental variable in this paper. Meanwhile, to deal with the possible endogeneity problem caused by the one-period lag of the dependent variables in the dynamic panel model, the benchmark model is estimated again in this paper by using systematic generalized moment estimation (SYS-GMM). 

Table 4 shows the results of robustness test. Models 1 to 2 show that GVC embedment has a significant suppressive effect on the ICEEE whether replacing dependent variables or dividing the sample into low, medium and high groups; the higher the degree of GVC embedment, the more significant the suppressive effect. Furthermore, Model 3 indicates that the emissions reduction coefficient of GVC embedment is still significantly negative after considering the possible endogeneity problem of the model. The robustness tests indicate that the results of this paper are robust.

### 6.4. Mechanism Test Results

To examine three paths of the impact of GVC embedment on the ICEEE, we construct the mediating effects model. Table 5 shows the mechanism test results that GVC embedment reduces the ICEEE by reducing energy consumption intensity, optimizing energy consumption structure and strengthening carbon emissions transfer; that is to say, GVC embedment will give play to the energy conservation effect, structure effect and transfer effect and make great contributions to the achievement of carbon emissions reduction targets.

#### 6.4.1. Energy Conservation Effect

Model 1 shows the results of energy conservation effect. Column 1 shows a significantly negative coefficient of the effect of GVC embedment on the mediating factor (EY), and column 3 shows the positive effect of energy consumption intensity on the ICEEE. Meanwhile, the coefficients $\beta_{2}\gamma_{3}$ and $\alpha_{2}$ have the same sign, indicating a significant partial mediating effect of energy consumption intensity in the effect of GVC embedment on the ICEEE. Furthermore, the quadratic term is introduced for regression. Column 2 shows that there is an inverted U-shaped relationship between GVC embedment and energy consumption intensity. Combining the regression results from columns 1 to 3, GVC embedment has an inverted U-shaped effect on the ICEEE through the energy conservation effect, which partially verifies the Hypothesis 2. The above results indicate that at the initial stage of GVC embedment, it does not reduce energy consumption per unit of output to reduce carbon emissions in exports, mainly because the industries initially embedding in GVC will increase their production and trade to maintain their position in GVC, and their energy consumption will continue to grow during a period of time. When the industry gradually increases its degree of GVC embedment, it will pay more attention to optimizing factor allocation and reducing the proportion of energy factor inputs, which will have a suppressive effect on the ICEEE.

#### 6.4.2. Structure Effect

Model 2 shows the results of structure effect. Column 1 shows a positive and insignificant coefficient of the effect of GVC embedment on the mediating factor (ES), which is not consistent with the theoretical expectation and further introduces a quadratic term for regression. Column 2 shows that there is an inverted U-shaped relationship between GVC embedment and energy consumption structure. Column 3 shows the negative effect of energy consumption structure on the ICEEE. Furthermore, by introducing the quadratic term, the $\beta_{2}\gamma_{3}$ coefficients and $\alpha_{2}$ have the same sign, indicating a significant partial mediating effect of energy consumption structure in the effect of GVC embedment on the ICEEE. Combining the regression results from columns 1 to 3, GVC embedment has an inverted U-shaped effect on the ICEEE through structure effect, which partially verifies the Hypothesis 3. The above results indicate that at the early stage of GVC embedment, it does not effectively optimize the energy consumption structure and even leads to a continuous increase in fossil energy sources such as coal and crude oil, which will increase domestic carbon emissions. The proper reason is that industries usually give priority to optimizing low-energy-consumption parts such as R&D, sales and management instead of traditional high-energy-consumption parts at the early stage of GVC embedment. As the degree of GVC embedment increases, structure effect plays a key role in reducing the ICEEE.

#### 6.4.3. Transfer Effect

Model 3 shows the results of transfer effect. Column 1 shows a significantly negative coefficient of the effect of GVC embedment on the mediating factor (FR), which is not consistent with the theoretical expectation, and the quadratic term is further introduced for regression. Column 2 shows that there is an inverted U-shaped relationship between GVC embedment and carbon emissions transfer. Column 3 shows the negative effect of carbon emissions transfer on the ICEEE. Furthermore, by introducing the quadratic term, the $\beta_{2}\gamma_{3}$ coefficients and $\alpha_{2}$ have the same sign, indicating a significant partial mediating effect of carbon emissions transfer in the effect of GVC embedment on the ICEEE. Combining the regression results from columns 1 to 3, GVC embedment has an inverted U-shaped effect on the ICEEE through the transfer effect, which partially verifies the Hypothesis 4. The above results also indicate that at the early stage of GVC embedment, countries may not effectively transfer domestic carbon emissions; as the degree of GVC embedment increases, the transfer effect starts to play a significant role, which will reduce domestic carbon emissions embodied in exports.

## 7. Conclusions

Based on the data of 17 manufacturing sectors in 36 economies from 2000 to 2016, this paper analyzes and empirically tests the effect and mechanism of GVC embedment on the intensity of carbon emissions embodied in exports (ICEEE). The three main conclusions are drawn as follows.

First, GVC embedment significantly reduces the ICEEE and promotes carbon emissions reduction, which is beneficial to realize the Sustainable Development Goals (SDGs) for countries around the word.

Second, GVC embedment not only directly promotes the carbon emissions reduction of exports but also reduces the ICEEE through three indirect paths which are reducing energy consumption intensity, optimizing energy consumption structure and enhancing carbon emissions transfer, respectively.

Third, the carbon emissions reduction effect of GVC embedment has obvious region, industry and embedment methods heterogeneity. Specifically, the inhibitory effect of GVC embedment on the ICEEE in developed countries is greater than that of developing countries. GVC embedment hinders ICEEE in low-tech manufacturing industries while promotes it in high-tech manufacturing industries, conversely. The forward embedment and complex embedment of GVC significantly reduce the ICEEE, while the inhibitory effect of the backward embedment of GVC is not significant, and the simple embedment of GVC even shows a promoting effect.

## 8. Discussions and Limitations

### 8.1. Contributions and Implications

This paper has two main contributions. First, the relationship between two dimensions of GVC embedment and carbon emissions embodied in exports is studied. It is found that GVC embedment is negatively related to carbon emissions embodied in exports, which is consistent with the purpose of the SDGs. This result provides theoretical support for manufacturing sectors of countries around the world to participate in GVC. Secondly, this paper reveals three indirect impact paths of GVC embedment on carbon emissions embodied in exports which are called the energy conservation effect, structure effect and transfer effect. This not only expands the relevant study of GVC and carbon emissions but also allows us to a new theoretical view and combines GVC embedment with energy conservation and carbon emissions reduction.

The conclusions of this paper also have certain practical implications to help policymakers design policies to reduce carbon emissions embodied in exports. First, GVC embedment can help promote carbon emissions reduction embodied in exports, especially for developing economies, which should more closely combine participation in the international division of labor with the goal of energy conservation and emissions reduction. Therefore, with the continued extension and refinement of GVC, governments should make effort to encourage enterprises to embed in GVC and climb to the high position. Second, GVC embedment can reduce carbon emissions embodied in exports by reducing energy consumption intensity, optimizing energy consumption structure and enhancing carbon emissions transfer. Therefore, on one hand, government should coordinate the formulation of energy conservation and emissions reduction policies, drive emissions reduction with energy conservation, encourage enterprises to benchmark international environmental standards, actively optimize the factor input and product export structure, accelerate the replacement of non-clean energy sources such as coal and crude oil by clean energy sources such as hydropower, wind power and photoelectricity and improve enterprises’ low-carbon competitiveness; on the other hand, it is beneficial to actively undertake the transfer of low-pollution international service industries and improve the service level of manufacturing. Finally, compared with high-tech manufacturing industries, the emissions reduction effect of GVC embedment on low-tech manufacturing is more significant; compared with simple embedment and backward embedment of GVC, complex embedment and forward embedment significantly reduce the intensity of carbon emissions embodied in manufacturing exports. Therefore, governments should actively guide low-tech industries and industries in the downstream position of GVC to improve their innovation level, technology absorption and transformation capacity and promote them to climb to the upstream position of GVC so as to further exert the export emissions reduction effect of GVC embedment and promote the green and low-carbon development of countries.

### 8.2. Limitations and Future Study Directions

Although its contributions notwithstanding, there are some limitations in this paper, which may suggest directions for further study. Firstly, this paper characterizes GVC embedment by its participation index based on the value-added trade perspective, which has some limitations. In the future study, GVC embedment can be measured by combining value-added with production stages perspectives. Secondly, this paper only explores the mechanism of the impact of GVC embedment on the carbon emissions of manufacturing embodied in exports, and further study on agriculture and service industries is needed. Finally, the continued spread of the COVID-19 has cast a shadow on the global economic development. Therefore, the future study may focus on the relationship between global supply chains and carbon emissions embodied in trade under this background.

## Figures and Tables

**Table 1:** Decompositions of value-added and carbon emissions embodied in exports.

Gross exports decomposition	Domestic value-added of exports (DVA)	Domestic value-added in exports of final products (DVA_FIN)	Domestic carbon emissions consumed by importing countries embodied in exports of final products (EEX_C1)	Domestic carbon emissions embodied in exports (EEX)	Carbon emissions embodied in exports decomposition
		Domestic value-added of intermediate exports to direct importing countries (DVA_INT)	Domestic carbon emissions consumed by importing countries embodied in exports of intermediate products (EEX_C2)		
		Domestic value-added of intermediate goods exported to direct importing countries and then exported to third countries (DVA_INT_REX)	Domestic carbon emissions consumed by third countries embodied in exports of intermediate products (EEX_C3)		
		Domestic value-added of intermediate exports returned and absorbed by the country (DVA_RDV)	Domestic carbon emissions consumed by the country embodied in exports of intermediate products (EEX_C4)		
	Foreign Value-added of exports (FVA)	Value-added of final products exports to importing Countries (FVA_FIN)	Carbon emissions from importing countries embodied in exports of final products (FEX_C1)	Foreign carbon emissions embodied in exports (FEX)	
		Importers’ value-added of intermediate products exports (FVA_INT)	Carbon emissions from importing countries embodied in exports of intermediate products (FEX_C2)		
		Third countries value-added of final products exports (FVA_FIN_REX)	Carbon emissions from third countries embodied in exports of final products (FEX_C3)		
		Third countries value-added of intermediate products exports (FVA_INT_REX)	Carbon emissions from third countries embodied in exports of intermediate products (FEX_C4)		

Note: Value-added and carbon emissions embodied in exports decompositions refer to the WWZ method and Meng et al. [56], respectively.

**Table 2:** Baseline regression results.

	Model 1	Model 2	Model 3	Model 4	Model 5	Model 6
*VARIABLES*	OLS	FE	REGH	D-REGH	D-REGH	D-REGH
*L.TEC*				0.753 *** (124.58)	0.741 *** (89.80)	0.721 *** (81.32)
*GVC_* Pat	0.223 *** (8.68)	−0.216 *** (−4.87)	−0.216 *** (−4.87)	−0.075 *** (−2.68)		
*Low × GVC_* Pat					−0.006 (−0.21)	
*High × GVC_* Pat						−0.249 *** (−3.37)
GVC_Pos	0.004 (0.10)	0.241 *** (3.83)	0.241 *** (3.83)	0.086 ** (2.17)	−0.012 (−0.36)	0.339 *** (3.15)
*TS*	−0.043 *** (−24.55)	−0.034 *** (−15.45)	−0.034 *** (−15.45)	−0.012 *** (−8.90)	−0.003 ** (−2.56)	−0.034 *** (−10.82)
*EMP*	0.039 *** (28.39)	0.022 *** (8.17)	0.022 *** (8.17)	0.010 *** (5.68)	0.004 *** (3.06)	0.030 *** (7.76)
*ES*	−0.001 ** (−2.30)	−0.006 *** (−6.91)	−0.006 *** (−6.91)	−0.002 *** (−3.59)	−0.001 (−1.53)	−0.006 *** (−3.87)
*OPC*	0.001 *** (11.85)	−0.001 *** (−8.59)	−0.001 *** (−8.59)	−0.000 *** (−3.30)	0.000 (0.29)	−0.000 * (−1.92)
*OPEN*	−0.092 *** (−8.34)	0.028 ** (2.54)	0.028 ** (2.54)	0.011 (1.60)	0.006 (1.14)	0.057 ** (2.22)
*TFP*	−0.030 *** (−6.15)	0.023 *** (4.56)	0.023 *** (4.56)	0.006 * (1.93)	−0.006 ** (−2.12)	0.029 *** (4.95)
*lnGDP*	−0.002 (−1.41)	−0.127 *** (−21.80)	−0.127 *** (−21.80)	−0.061 *** (−16.46)	−0.052 *** (−17.57)	−0.095 *** (−9.84)
*FDIR*	−0.069 *** (−15.73)	−0.024 *** (−3.43)	−0.024 *** (−3.43)	−0.015 *** (−3.37)	−0.014 ** (−2.13)	−0.007 (−1.15)
*Constant*	0.106 ** (2.17)	2.064 *** (19.78)	1.712 *** (17.66)	0.715 *** (11.59)	0.540 *** (9.77)	1.261 *** (7.85)
*Country Effect*	NO	YES	YES	YES	YES	YES
*Industry Effect*	NO	YES	YES	YES	YES	YES
*Time Effect*	NO	YES	YES	YES	YES	YES
*Observations*	10,352	10,352	10,352	10,350	5175	5175
*R-squared*	0.254	0.548	0.548	0.820	0.883	0.807

Note: ***, **, * denote 1%, 5%, and 10% significance levels, respectively, and *t* values are in parentheses.

**Table 3:** Heterogeneity regression results.

	Model 1		Model 2	
*VARIABLES*	Developed Countries	Developing Countries	Low-Tech Industries	High-Tech Industries
*L.TEC*	0.751 *** (103.97)	0.701 *** (56.10)	0.760 *** (101.11)	0.655 *** (59.53)
*GVC_* Pat	−0.285 *** (−5.12)	−0.082 ** (−2.12)	−0.099 ** (−2.55)	−0.011 (−0.31)
*Constant*	1.129 *** (9.82)	1.049 *** (10.27)	0.754 *** (8.76)	0.621 *** (7.81)
*Control Variables*	YES	YES	YES	YES
*Country Effect*	YES	YES	YES	YES
*Industry Effect*	YES	YES	YES	YES
*Time Effect*	YES	YES	YES	YES
*Observations*	7800	2549	6696	3653
*R-squared*	0.804	0.853	0.820	0.822
	Model 3			
	Embedment position		Number of cross-border	
*VARIABLES*	Forward embedment	Backward embedment	Simple embedment	Complex embedment
*L.TEC*	0.753 *** (124.42)	0.754 *** (124.74)	0.753 *** (124.52)	0.753 *** (124.59)
*GVC_* Pat	−0.161 *** (−3.80)	−0.031 (−0.49)	0.030 ** (1.99)	−0.074 *** (−3.63)
*Constant*	0.723 *** (14.31)	0.602 *** (8.81)	0.546 *** (15.90)	0.557 *** (17.55)
*Control Variables*	YES	YES	YES	YES
*Country Effect*	YES	YES	YES	YES
*Industry Effect*	YES	YES	YES	YES
*Time Effect*	YES	YES	YES	YES
*Observations*	10,350	10,350	10,350	10,350
*R-squared*	0.820	0.820	0.820	0.820

Note: ***, ** denote 1% and 5% significance levels, respectively, and *t* values are in parentheses.

**Table 4:** Robustness test results.

*VARIABLES*	Model 1	Model 2			Model 3		
					IV-2SLS	IV-DREGH	SYS-GMM
*L.TEC*		0.687 *** (64.06)	0.616 *** (54.84)	0.732 *** (66.16)	0.740 *** (106.97)	0.805 *** (60.18)	0.347 *** (3.52)
*L.TECT*	0.793 *** (142.79)						
*GVC_* Pat	−0.0002 ** (−2.44)				−1.613 *** (−14.44)	−1.370 *** (−4.34)	−5.495 *** (−2.84)
*GVC_*Pat × *D1*		−0.046 (−1.44)					
*GVC_*Pat × *D2*			−0.024 (−0.43)				
*GVC_*Pat × *D3*				−0.306 *** (−2.66)			
*Constant*	0.016 *** (10.94)	0.789 *** (12.45)	1.125 *** (9.11)	1.793 *** (7.04)	3.862 *** (17.73)		
*Control Variables*	YES	YES	YES	YES	YES	YES	YES
*Country Effect*	YES	YES	YES	YES	YES	YES	YES
*Industry Effect*	YES	YES	YES	YES	YES	YES	YES
*Time Effect*	YES	YES	YES	YES	YES	YES	YES
*AR(2)*							0.752
*Hansen-p*							0.374
*Observations*	10,351	3446	3453	3451	10,350	10,350	9145
*R-squared*	0.850	0.8893	0.8384	0.8069	0.767	0.683	

Note: ***, ** denote 1% and 5% significance levels, respectively, and *t* values are in parentheses.

**Table 5:** Mechanism test results.

	Model 1			Model 2			Model 3		
	Column 1	Column 2	Column 3	Column 1	Column 2	Column 3	Column 1	Column 2	Column 3
*VARIABLES*	EY	EY	TEC	ES	ES	TEC	FR	FR	TEC
*L.TEC*			0.752 *** (124.14)			0.753 *** (124.20)			0.730 *** (117.20)
*L.Med*	0.814 *** (175.50)	0.806 *** (171.61)		0.868 *** (221.94)	0.867 *** (221.18)		0.675 *** (122.13)	0.694 *** (132.21)	
*GVC_* Pat	−0.084 *** (−2.86)	−0.267 *** (−7.67)	−0.068 ** (−2.44)	0.029 (0.47)	0.145 ** (2.00)	−0.076 *** (−2.71)	−0.077 *** (−3.30)	−0.331 *** (−7.70)	−0.104 *** (−3.72)
${\left(GVC\_Pat\right)}^{2}$		0.109 *** (9.63)			−0.070 *** (−2.98)			0.062 *** (4.50)	
*Med*			0.020 *** (4.21)			0.003 * (1.72)			−0.107 *** (−13.61)
*Constant*	0.116 * (1.81)	0.253 *** (3.88)	0.717 *** (11.62)	−0.002 (−0.01)	−0.093 (−0.68)	0.724 *** (11.69)	0.133 *** (2.62)	0.777 *** (9.56)	0.820 *** (13.30)
*Control Variables*	YES	YES	YES	YES	YES	YES	YES	YES	YES
*Country Effect*	YES	YES	YES	YES	YES	YES	YES	YES	YES
*Industry Effect*	YES	YES	YES	YES	YES	YES	YES	YES	YES
*Time Effect*	YES	YES	YES	YES	YES	YES	YES	YES	YES
*Observations*	10,351	10,351	10,350	10,333	10,333	10,333	10,351	10,352	10,350
*R-squared*	0.900	0.901	0.820	0.907	0.907	0.820	0.942	0.859	0.823

Note: ***, **, * denote 1%, 5%, and 10% significance levels, respectively, and *t* values are in parentheses.

## Data Availability

The data that support the findings of this study are openly available in CNKI at http://cnki.nbsti.net/CSYDMirror/area/home/index/D26 (accessed on 1 December 2022).

## References

[1] Liu H., Zong Z., Hynes K., De Bruyne K. (2020). Can China reduce the carbon emissions of its manufacturing exports by moving up the global value chain?. Res. Int. Bus. Financ..

[2] Liu Y.L., Li Z.H., Yin X.M. (2018). Environmental Regulation, Technological Innovation and Energy Consumption-A Cross-Region Analysis in China. J. Clean. Prod..

[3] Song M.L., Wang S.H. (2017). Participation in Global Value Chain and Green Technology Progress: Evidence from Big Data of Chinese Enterprises. Environ. Sci. Pollut. Res..

[4] Huang H., Zhang Z., Jiang F. (2022). Environmental effects of global value chain embedding in manufacturing industry in countries along the Belt and Road. Front. Env..

[5] Ball D.S. (1968). Trade Liberalization Among Industrial Countries: Objectives and Alternatives. Political Sci. Rev..

[6] Scott M.F.G. (1975). Intra-Industry Trade: The Theory and Measurement of International Trade in Differentiated Products. Econ. J..

[7] Findlay R. (1978). An “Austrian” model of international trade and interest rate equalization. J. Political Econ..

[8] Porter M. (1985). Competitive Advantage: Creating and Sustaining Superior Performance.

[9] Krugman P., Venables A.J. (1995). Globalization and the Inequality of Nations. Q. J. Econ..

[10] Sturgeon T.J. (2001). How do we define value chains and production networks?. IDS Bull..

[11] Gereffi G., Korzeniewicz M. (1994). Commodity Chains and Global Capitalism.

[12] Hummels D., Ishii J., Yi K.M. (2001). The nature and growth of vertical specialization in world trade. J. Int. Econ..

[13] Daudin G., Rifflart C., Schweisguth D. (2011). Who produces for whom in the world economy?. Can. J. Econ. Rev. Can. D’économique.

[14] Johnson R.C., Noguera G. (2012). Accounting for intermediates: Production sharing and trade in value added. J. Int. Econ..

[15] Fally T. (2012). Production Staging: Measurement and Facts.

[16] Antràs P., Chor D., Fally T., Hillberry R. (2012). Measuring the upstreamness of production and trade flows. Am. Econ. Rev..

[17] Antràs P., Chor D. (2013). Organizing the global value chain. Econometrica.

[18] Koopman R., Wang Z., Wei S. (2014). Tracing Value-added and Double Counting in Gross Exports. Am. Econ. Rev..

[19] Wang Z., Wei S.J., Zhu K.F. (2013). Quantifying International Production Sharing at the Bilateral and Sector Levels.

[20] Wang Z., Wei S.J., Zhu K.F. (2017). Characterizing Global Value Chains: Production Length and Upstreamness.

[21] Wang Z., Wei S.J., Zhu K.F. (2017). Measures of Participation in Global Value Chains and Global Business Cycles.

[22] Zhao Y.H., Liu Y. (2011). Implicit carbon measurement of Chinese export products based on input-output method. China Popul.-Resour. Environ..

[23] Qiao S.Y., Li Z.Y., Xiang N. (2018). Tracing the implied carbon emission flow of intermediate goods trade and comparison of multi-regional input-output databases—A study based on WIOD, Eora, and EXIOBASE data. Financ. Trade Econ..

[24] Tang Y.D., Zhu S.J., Luo Y. (2022). Input servitization, global value chain, and carbon mitigation: An input-output perspective of global manufacturing industry. Econ. Model..

[25] Cheng B.D., Li H.J. (2020). Accounting for implied carbon emissions of “Belt and Road” exports from the perspective of value-added trade. Seeking.

[26] Li Y.M., Fu J.F. (2010). Structural decomposition analysis of implied carbon emission growth in China’s export trade. China Popul.-Resour. Environ..

[27] Yin W.H. (2019). A study on the change of implied carbon emission intensity of China’s export trade and the driving factors—Based on CMRIO-SDA model. Explor. Econ. Issues.

[28] Wang Y., Wei B.Y. (2011). Implicit carbon decomposition of China’s international trade based on LMDI method. China Popul.-Resour. Environ..

[29] Li S.S., Luo L.W. (2012). The impact of FDI industry structure on implied carbon emissions of China’s foreign trade—An empirical analysis based on the decomposition of index factors. Resour. Sci..

[30] Qian Z.Q., Yang L.K. (2016). Study on the impact of vertical division of labor in East Asia on implied carbon in China’s foreign trade—A cross-period comparison based on MRIO-SDA method. Resour. Sci..

[31] Fu Z.H. (2018). Discerning the implied problem calculation method based on input-output table--to calculate the implied domestic carbon emission intensity of China’s value added export as an example. Res. Quant. Econ. Technol. Econ..

[32] Li Q., Wu S., Li S. (2022). Weighing China’s Embodied CO2 Emissions and Value Added under Global Value Chains: Trends, Characteristics, and Paths. J. Environ. Manag..

[33] Wang W.J., Xiang Q.F. (2011). Accounting for implied carbon emissions and responsibility allocation in international trade. China Ind. Econ..

[34] Yan Y.F., Zhao Z.X. (2012). A study on the measurement of implied carbon in China’s foreign trade—A perspective based on the definition of carbon emission responsibility. Int. Trade Issues.

[35] Liu Y., Chen S., Chen B. (2017). Analysis of CO_2_ emissions embodied in China’s bilateral trade: A non-competitive import input-output approach. J. Clean. Prod..

[36] Meng F.X., Su M.R., Hu Y.C. (2019). Implicit carbon transfer in trade of China and typical countries along the “Belt and Road”. China Popul.-Resour. Environ..

[37] Wang S., Zhao Y. (2019). Carbon emissions embodied in China–Australia trade: A scenario analysis based on input-output analysis and panel regression models. J. Clean. Prod..

[38] Li J., Liu Y. (2020). Trade impacts on embodied carbon emissions—Evidence from the bilateral trade between China and Germany. Int. J. Environ. Res. Public Health.

[39] Lv Y., Lv Y.L. (2019). Analysis of environmental effects of China’s participation in global value chains. China Popul.-Resour. Environ..

[40] Zhao Y.H., Zheng L., Liu X.C. (2021). A study on the impact of global value chain embedment on implied carbon in China’s export trade. Int. Trade Issues.

[41] Chen H., Zhang C., Yin K. (2022). The Impact of Global Value Chain Embedding on Carbon Emissions Embodied in China’s Exports. Front. Environ. Sci..

[42] Lv Y.F., Cui X.H., Wang D. (2019). Global value chain participation and trade implied carbon. Quant. Econ. Tech. Econ. Res..

[43] Pan A. (2017). The impact of global value chain division of labor on China’s implied carbon emissions in foreign trade. Int. Econ. Trade Explor..

[44] Wang Z., Zhang Y., Liao C., Ai H., Yang X. (2022). What Contributes to the Growth of China’s Embodied CO_2_ Emissions? Incorporating the Global Value Chains Concept. Appl. Econ..

[45] Liu S.G., Wu H.Y., Ma T., Zhang L., Peng L., Yu J.X. (2015). Using global value chains to promote industrial upgrading. Int. Econ. Rev..

[46] Su H., Zheng L., Mou Y.F. (2017). Factor endowment and industrial upgrading of Chinese manufacturing industry—An analysis based on WIOD and Chinese industrial enterprise database. Manag. World.

[47] Lv Y., Huang Y.X. (2017). Productivity effects of global value chain embedment:impact and mechanism analysis. World Econ..

[48] Gereffi G. (2013). A global value chain perspective on industrial policy and development in emerging markets. Duke J. Comp. Int. Law.

[49] Sheng B., Chen S. (2015). How global value chains have changed trade policy:implications and insights for industrial upgrading. Int. Econ. Rev..

[50] Bai J.H., Yu X.W. (2022). The impact of global value chain embedment on energy saving and emission reduction: Theory and empirical evidence. Financ. Trade Econ..

[51] Akhmat G., Zaman K., Shukui T., Irfan D., Khan M.M. (2014). Does energy consumption contribute to environmental pollutants? Evidence from SAARC countries. Environ. Sci. Pollut. Res..

[52] Wang S.Y., Zheng L.K. (2019). The impact of global value chain embedment characteristics on the differentiation of technological complexity of exports. Quant. Econ. Tech. Econ. Res..

[53] Sultani, Sheng B. (2020). Interactive effects of global value chains, localized industrial agglomeration and firm productivity. Econ. Res..

[54] Liu B., Wang N.J., Yu M.J. (2021). Implied carbon in manufacturing service factor inputs and exports—A study based on the perspective of environmental cost of global value chain. J. Renmin Univ. China.

[55] Copeland B., Taylor M.S. (1997). A Simple Model of Trade, Capital Mobility, and the Environment.

[56] Meng B., Peters G.P., Wang Z., Li M. (2018). Tracing CO_2_ emissions in global value chains. Energy Econ..

[57] Correia S. (2016). A Feasible Estimator for Linear Models with Multi-Way Fixed Effects. http://scorreia.com/research/hdfe.pdf.

[58] Wen Z.L., Zhang L., Hou J.T. (2004). Mediation effect test procedure and its application. J. Psychol..

[59] Peng S.J., Zhang W.C., Sun C.W. (2015). Research on carbon emission measurement and influencing factors on production side and consumption side in China. Econ. Res..

[60] Xu J.Y., Mao Q.L. (2016). Market survival analysis of Chinese firms: Do imports of intermediate goods matter?. Financ. Res..

[61] Huang L.Y., Xie H.Q., Liu D.D. (2017). Technological progress path selection and implied carbon emission intensity of China’s manufacturing exports. China Popul.-Resour. Environ..

